# Simultaneous Assessment of mTORC1, JAK/STAT, and NLRP3 Inflammasome Activation Pathways in Patients with Sarcoidosis

**DOI:** 10.3390/ijms241612792

**Published:** 2023-08-14

**Authors:** Raisa Kraaijvanger, Carmen A. Ambarus, Jan Damen, Joanne J. van der Vis, Karin M. Kazemier, Jan C. Grutters, Coline H. M. van Moorsel, Marcel Veltkamp

**Affiliations:** 1 Interstitial Lung Diseases Center of Excellence, Department of Pulmonology, St. Antonius Hospital, 3435 CM Nieuwegein, The Netherlands; r.kraaijvanger@antoniusziekenhuis.nl (R.K.);; 2 Interstitial Lung Diseases Center of Excellence, Pathologie DNA, Department of Pathology, St. Antonius Hospital, 3435 CM Nieuwegein, The Netherlands; 3 Pathologie DNA, Department of Pathology, Jeroen Bosch Hospital, 5223 GZ ‘s-Hertogenbosch, The Netherlands; 4 Department of Clinical Chemistry, St Antonius ILD Center of Excellence, St. Antonius Hospital, 3435 CM Nieuwegein, The Netherlands; 5 Center of Translational Immunology, University Medical Center Utrecht, 3508 GA Utrecht, The Netherlands; 6 Division of Heart and Lungs, University Medical Center Utrecht, 3508 GA Utrecht, The Netherlands

**Keywords:** signaling pathways, sarcoidosis, phenotyping, granuloma

## Abstract

The unknown etiology of sarcoidosis, along with the variability in organ involvement and disease course, complicates the effective treatment of this disease. Based on recent studies, the cellular inflammatory pathways involved in granuloma formation are of interest regarding possible new treatment options, such as the mechanistic (formerly mammalian) target of rapamycin complex 1 (mTORC1) pathway, the Janus kinase/signal transducers and activators of transcription (JAK/STAT) pathway, and the nucleotide-binding domain, leucine-rich-containing family, pyrin domain-containing-3 (NLRP3) inflammasome pathway. The aim of this study was to explore the potential coexpression of these three inflammatory pathways in patients with sarcoidosis and see whether possible differences were related to disease outcome. The tissue of 60 patients with sarcoidosis was used to determine the activity of these three signaling pathways using immunohistochemistry. The activation of NLRP3 was present in 85% of all patients, and the activation of mTORC1 and JAK/STAT was present in 49% and 50% of patients, respectively. Furthermore, the presence of NLRP3 activation at diagnosis was associated with a chronic disease course of sarcoidosis. Our finding of different new conceptual inflammatory tissue phenotypes in sarcoidosis could possibly guide future treatment studies using the available inhibitors of either NLRP3, JAK-STAT, and mTORC1 inhibitors in a more personalized medicine approach.

## 1. Introduction

Sarcoidosis is a heterogeneous multisystem inflammatory disease characterized by noncaseating granuloma formation of an unknown cause [1]. Although sarcoidosis resolves in most cases within 2–3 years, in about 30% of patients, inflammation persists and leads to a chronic, sometimes progressive, and even fibrotic, disease. For this group of patients, systemic treatment is required [1,2,3]. Recently, an updated version of the treatment guideline for sarcoidosis was published, suggesting a step-up approach in the management of pulmonary sarcoidosis using steroids, DMARDs, and TNF-alpha inhibitors, respectively [4]. In the case of therapy refractory sarcoidosis, guidance on fourth-line therapy is lacking.

Based on recent studies, three key cellular inflammatory pathways involved in granuloma formation are of interest regarding possible new treatment options [5]. These key pathways include the mechanistic (formerly mammalian) target of rapamycin complex 1 (mTORC1) pathway, the Janus kinase/signal transducers and activators of transcription (JAK/STAT) pathway, and the nucleotide-binding domain, leucine-rich-containing family, pyrin domain-containing-3 (NLRP3) inflammasome pathway. The role of the mTORC1 pathway in the pathogenesis of sarcoidosis was first described by Linke et al. [6], who showed that activation of the mTORC1 signaling pathway in macrophages resulted in granuloma formation similar to sarcoidosis in mice by staining it with the downstream target phosphor-S6 ribosomal protein (p-S6). In addition, they identified activation of this pathway in one third of granulomas obtained from 27 biopsies from sarcoidosis patients. Data from our own group confirmed these findings with the activation of the mTORC1 pathway seen in 43% of 74 Dutch sarcoidosis patients [7]. Furthermore, Linke et al. [6] reported that by treating mice with everolimus, an mTOR inhibitor, the granuloma in the mice completely resolved. Based on these results, it was questioned whether treatment with an mTOR inhibitor would also be beneficial in sarcoidosis patients. Successful treatment with sirolimus was already reported in a patient with pulmonary sarcoidosis [8], and a pilot study with sirolimus in cutaneous sarcoidosis is ongoing (ClinicalTrials.gov, Identifier: NCT05458492).

Damsky et al. [9] were the first to describe the role of the JAK/STAT signaling pathway in sarcoidosis. Using RNA sequencing, they showed that significantly higher levels of STAT1 and STAT3 were found in patients with cutaneous sarcoidosis compared to healthy controls. STAT1 and STAT3 play a role in type 1 T-helper (Th1) and Th17 cell differentiation and are implicated in the pathogenesis of sarcoidosis [10]. Recently, Damsky et al. [11] reported that treatment with tofacitinib, a JAK inhibitor, resulted in a decrease in cutaneous lesions in patients with sarcoidosis. Furthermore, positive treatment effects using tofacitinib have been described in patients with pulmonary sarcoidosis [12,13]. However, in order to determine the exact role of the JAK/STAT signaling pathway in sarcoid pathogenesis, further investigation is required.

Huppertz et al. [14] suggested a role for NLRP3 inflammasome in sarcoidosis pathogenesis by identifying the activation of IL-1β and caspase-1 in the granulomatous tissue of sarcoidosis patients. In addition, they showed that positive staining for IL-1β correlated with a progressive course of disease [14]. As with the other two key pathways, the NLRP3 inflammasome could also be a potential target for treatment in sarcoidosis. A placebo controlled double-blind trial assessing the effectiveness of canakinumab, which neutralizes the biological activity of IL-1β, in pulmonary sarcoidosis was completed in 2019, but the results are not (yet) published (ClinicalTrials.gov, Identifier: NCT02888080).

Different studies have been published addressing one of the three abovementioned inflammatory pathways; however, data on coexistence in a heterogeneous disease like sarcoidosis are lacking. In order to explore the potential coexpression of these three inflammatory pathways in patients with sarcoidosis, we conducted this study. We hypothesized that there will be differences between the activation of these three pathways in individual patients. Furthermore, we assessed whether the presence or differences in these inflammatory pathways could be linked to disease outcome.

## 2. Results

### 2.1. Expression in Granulomatous Tissue

Formalin-fixed paraffin-embedded tissue blocks were available from 60 patients with sarcoidosis. The biopsies contained lung (*n* = 21), lymph nodes (*n* = 21), skin (*n* = 15), bone marrow (*n* = 2), and nasal concha (*n* = 1). The characteristics of the cohort are shown in Supplementary Table S1. There were no differences in the expression of the pathways between patients with and without therapy at time of biopsy.

**Figure 1:**
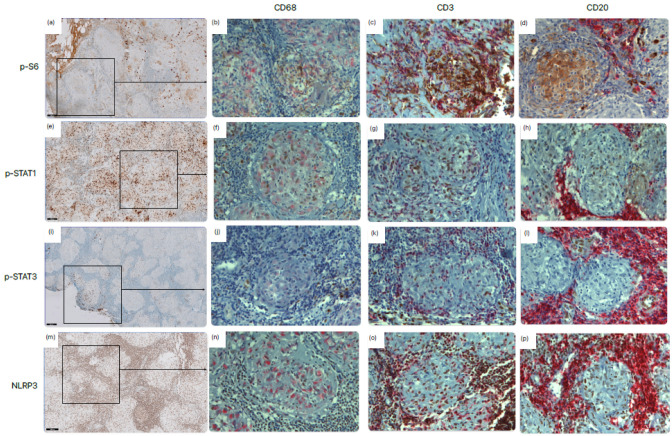
Expression of the mTORC1 (p-S6), JAK-STAT (p-STAT1 and p-STAT3), and NLRP3 (NLRP3) signaling pathways in granulomatous tissue samples from sarcoidosis patients. Lymph node tissue was immunohistochemically stained for (**a**–**d**) phosphor-S6 ribosomal protein (p-S6), (**e**–**h**) p-STAT1, (**i**–**l**) p-STAT3, and (**m**–**p**) NLRP3. This staining is shown in brown. In addition to the single-staining (**a**,**e**,**i**,**m**), double staining was used to show expression in macrophages (CD68) (**b**,**f**,**j**,**n**), T-cells (CD3) (**c**,**g**,**k**,**o**), and B-cells (CD20) (**d**,**h**,**l**,**p**). This staining is shown in red. Scale bars = 100 μm (**a**,**e**,**i**,**m**) and 20 μm (**b**–**d**,**f**–**h**,**j**–**l**,**n**–**p**).

Figure 1 shows the expression of the signaling pathways in the main cellular components of a granuloma, namely, macrophages, T-cells, and B-cells. Regarding mTORC1, it was seen that this pathway was active in macrophages, T-cells, and B-cells. For the JAK-STAT signaling pathway, it was seen that STAT1 was activated in macrophages and T- and B-cells, while STAT3 expression was only present in T- and B-cells. The NLRP3 inflammasome signaling pathway was activated in macrophages and T- and B-cells. The expression of the inflammatory signaling pathways differed between tissue sections (Supplementary Figure S1).

#### 2.1.1. Immunohistochemical Scoring

Based on the difference in staining intensity and the percentage of positive cells, the standardized immunohistochemical (IHC) score was obtained (Table 1). The scoring system is fully explained in Section 4.3. For all tissue sections, the overall mean IHC score for p-STAT3 was 0.45 ± 0.91; for p-S6, the mean IHC score was 1.03 ± 1.29; for p-STAT1, the mean IHC score was 1.38 ± 2.03; and for NLRP3, the mean IHC score was 4.27 ± 2.60. By subdividing the IHC score into three groups, negative (IHC = 0), weak (IHC = 1–4), and strong (IHC ≥ 5), we aimed to define the specific inflammatory phenotypes of sarcoidosis. Looking at the different groups, it was seen that for lymph node and skin biopsy tissue, the majority of the cases with a positive NLRP3 expression belonged to the groups with a high IHC (57.1% and 86.7%, respectively), while the NLRP3 expression in lung tissue was more equally divided between the three groups (*p* = 0.001). Also, for the STAT1 expression, a significant difference was observed for the IHC groups (*p* = 0.046), obtained by the fact that the majority (66.7%) of the skin tissue showed a positive expression of the JAK/STAT pathway. For NLRP3, a strong expression (IHC ≥ 5) was significantly seen more in patients with a history of smoking (*p* = 0.002), which was not observed for the other signaling pathways. There were no differences observed for medication use at time of biopsy in ethnicity nor sex. Furthermore, no difference occurred between the three IHC groups and the Scadding stages of sarcoidosis for any of the signaling pathways.

#### 2.1.2. Co-Occurrence of Signaling Pathways

**Figure 2:**
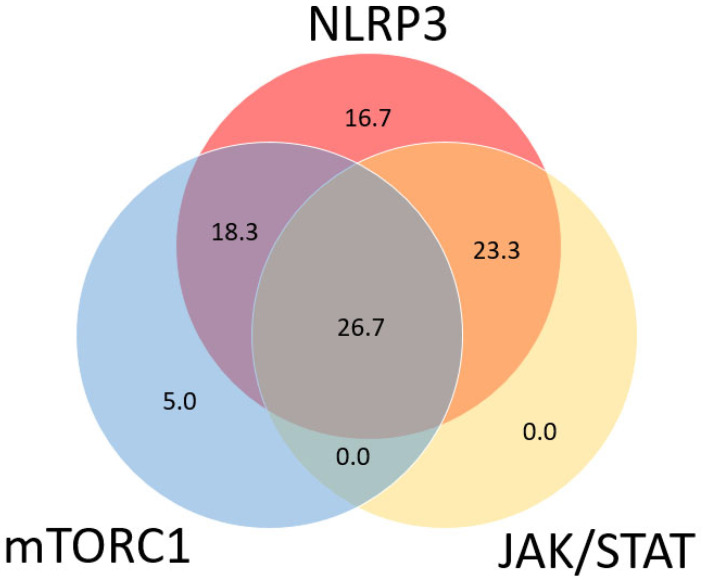
Venn diagram showing co-occurrence of active inflammatory signaling pathways in sarcoidosis patients (*n* = 60). Every circle represents one of the pathways, with overlapping sections representing tissue sections with a positive expression of two or more signaling pathways. Numbers represent percentage of patients with a positive expression of one or more signaling pathways. In total, 85.0% of patients were shown to be positive for the NLRP3 inflammasome signaling pathway compared to 50.8% positive for mTORC1 and 50.0% positive for JAK/STAT.

Figure 2 shows a Venn diagram of the distribution of the activation of the different signaling pathways in all 60 tissue sections. The NLRP3 signaling pathway was active in the majority of sarcoidosis patients (85.0%). The expression of p-STAT1 and p-STAT3 is further indicated as STAT expression, as no p-STAT3 expression was observed without the expression of p-STAT1. Of the 51 patients positive for NLRP3, 11 patients (21.6%) were also positive for mTORC1, 14 patients (27.5%) were also positive for STAT, and 16 patients (31.4%) were positive for both mTORC1 and STAT. Of the nine remaining sarcoidosis patients, three (5.0%) were shown to have a positive expression solely for the mTORC1 signaling pathway, and six (10%) were negative for all signaling pathways. Activation of the JAK/STAT signaling pathway was only observed in tissue sections positive for NLRP3. Looking at the clinical characteristics, it was seen that the group of patients positive for all three signaling pathways were significantly younger than the other groups, with a mean age of 36.3 ± 7.7 years at time of biopsy (*p* = 0.045). Furthermore, this group contained the highest percentage of ever smokers (81.5%); however, this was marginally insignificant (*p* = 0.070). There was no difference in the amount of positive signaling pathways between the different types of tissue material. The majority of patients treated with prednisone (66.7%) were shown to be positive for all three signaling pathways; however, this difference was also not significant. Furthermore, no difference was observed for the Scadding stage at time of biopsy nor for extra pulmonary involvement.

### 2.2. Potential Biomarker for Progressive Disease

**Figure 3:**
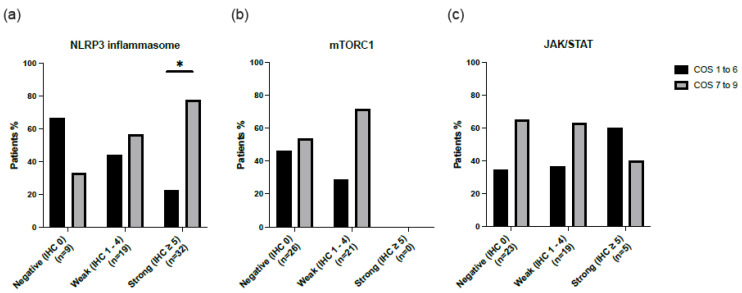
Clinical outcome score (COS) at five-year follow-up for sarcoidosis patients stained for three inflammatory signaling pathways: (**a**) NLRP3, (**b**) mTORC1, and (**c**) JAK/STAT. In the group stained for the NLRP3 inflammasome signaling pathway, significantly more patients with IHC ≥ 5 were shown to have a high COS (*p* = 0.021). * *p*-value < 0.05.

The disease course was determined using the clinical outcome score (COS) 5 years after biopsy. The COS was divided into two groups, low (COS 1–6) and high (COS 7–9), with COS ≥ 7 representing chronic disease requiring immunosuppressive therapy. For 47 patients, their clinical characteristics were available at 5-year follow-ups after disease diagnosis. Figure 3 shows the COS for all inflammatory signaling pathways. For the NLRP3 inflammasome pathway, patients with a strong IHC staining (IHC ≥ 5) had a significantly higher COS score (77.3% *p* = 0.021). This pattern was not observed in the mTORC1 nor the JAK/STAT signaling pathways. Furthermore, no significant difference was observed for the presence or absence of mTORC1 and/or JAK/STAT in cases positive for NLRP3 in relation to the COS score at the 5-year follow-up (*p* = 0.257).

### 2.3. Activity of Signaling Pathways in Broncho Alveolar Lavage Fluid (BALF)

Tissue samples are mainly taken during the diagnostic trajectory of patients with sarcoidosis, making it difficult to monitor the activity of the studied signaling pathways over time. A less invasive technique, which can be repeated more easily, is broncho alveolar lavage (BAL). In a different retrospective cohort of 25 patients with sarcoidosis in which BAL was performed during the same period as the biopsy, we assessed the inflammatory activity of the three pathways in both tissue and BAL fluid (BALF). The mean age of this cohort was 44.1 ± 8.3, and 64.0% were male. Five subjects (20.0%) had a history of smoking, and the majority (80.0%) were of Caucasian ethnicity. At the time of BAL, four (16.0%) patients received immunosuppressive treatment.

#### Correlation of Expression in BALF and Tissue

**Figure 4:**
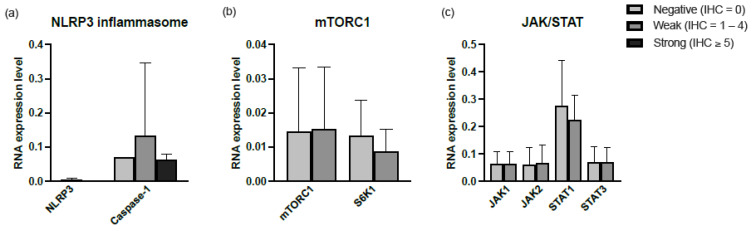
Relative mRNA expression levels in cells of BALF of (**a**) NLRP3 inflammasome, (**b**) JAK/STAT, and (**c**) mTORC1 signaling pathways. For every protein, the RNA expression levels were divided into 3 groups based on the IHC score. Abbreviations: NLRP3, NLR family pyrin domain-containing-3; JAK1, Janus kinase-1; JAK2, Janus kinase-2; STAT1, signal transducers and activators of transcription-1; STAT3, signal transducers and activators of transcription-3; mTORC1, mechanistic target of rapamycin complex 1; S6K1, target protein S6 kinase-1.

Tissue biopsies from lung (14 samples), lymph nodes (8 samples), and skin (3 samples) were stained for the inflammatory signaling pathways and were scored according to the IHC score. The mRNA expression in BALF was compared to the IHC staining in tissue. Figure 4 shows the average mRNA expression for each IHC group. No difference was observed for any of the signaling pathways. Furthermore, there was no correlation between the mRNA levels in BALF and IHC staining in tissue.

## 3. Discussion

Over the years, multiple studies have already described a possible role for the mTORC1, JAK/STAT, and NLRP3 inflammasome inflammatory pathways [5] in the pathogenesis of sarcoidosis. However, to our knowledge, these pathways have never been studied simultaneously in one cohort. In our study, we found that NLRP3, JAK-STAT, and mTORC1 are not simultaneously active in all patients’ sarcoidosis at diagnosis. Furthermore, the NLRP3 pathway seems most active in patients with sarcoidosis at diagnosis, and its presence is associated with worse clinical outcomes of the disease.

Histologically, sarcoidosis is characterized by well-formed granuloma consisting of arranged layers of immune cells, with a prominent core of macrophage aggregates accompanied by mainly T- and B-cells in an outer layer [15]. By staining macrophages and T- and B-cells, we were able to assess the inflammatory signaling pathways in these specific cells.

The inflammatory pathways studied were all active in macrophages and T-cells, as well as B-cells, except for STAT3. The activation of STAT3 was found in T-cells and B-cells but not in macrophages. Previously, Damsky et al. [9] already described p-STAT1 to be strongest in the center of the granuloma and more prominent in macrophages than in T-cells, while p-STAT3 was strongest in T-cells positioned around the granulomas. The replication of this finding is interesting due to the fact that STAT3 expression is normally seen in all mammalian cells and could suggest that aberrant STAT3 signaling plays a role in sarcoidosis disease pathogenesis, as is already suggested for inflammatory diseases like rheumatoid arthritis and cancer [16]. Also, animal models of psoriasis demonstrated that inhibition of the JAK/STAT3 pathway promoted macrophage polarization, which upregulated a profibrotic factor and, thereby, favorably alleviated tissue inflammation [17,18].

When comparing the presence of the three studied pathways, we found that compared to mTORC1 and STAT, the activation of the NLRP3 inflammasome pathway had both the highest prevalence (85%) and IHC score (4.27 ± 2.60) in patients with sarcoidosis at diagnosis. Within the group of NLRP3-positive patients, there was an equal distribution between the presence and/or absence of mTORC1 and/or JAK/STAT activation. Although confirming our initial hypothesis, an intriguing question remains why six different conceptual inflammatory phenotypes were found in a cohort of patients with sarcoidosis. We, therefore, correlated these inflammatory phenotypes with disease course and found that patients with a high tissue expression of NLRP3 at diagnosis had a worse clinical outcome, measured using the COS score. The observation that patients with active NLRP3 more often received a third-line treatment for sarcoidosis compared to patients without the activation of this pathway at diagnosis strengthens this association. Therefore, measuring the activity of the NLRP3 inflammasome signaling pathway in tissue might serve as a predictive biomarker for a more chronic disease course in patients with sarcoidosis. This was not observed for the other signaling pathways mTORC1 and JAK/STAT. Although very speculative, the association between NRLP3 activation and disease course, irrespective of mTORC1 and STAT activation, could suggest that inhibiting the inflammasome pathway might be more effective than the use of mTORC1- and STAT inhibitors in the treatment of sarcoidosis.

Previous studies reported the role of NLRP3 inflammasome activation in a profibrotic phenotype in systemic sclerosis, but it was found to be impaired in IPF BALF macrophages [19,20]. Due to the possible predictive value of NLPR3 activation in tissue and disease course, we investigated whether this signal would also correlate with the NLRP3 activation status in the alveolar compartment using BAL fluid in order to increase clinical applicability. No differences were found between NLRP3 activation in tissue and mRNA expression for neither NLRP3 nor caspase-1 in BAL fluid. In addition, no correlation between the mRNA expression levels in BALF and the activation in tissue for either mTORC1 or STAT was found. Our negative findings regarding mTORC1 are not in line with the previous data published by Prasse et al. [21]. In their recent study, they reported elevated levels of p-S6 in BAL cells of sarcoidosis patients with active disease and no immunosuppressive treatment. We measured mTORC1 and its direct downstream protein S6K1 in BAL cells. In its turn, p-S6 is activated by S6K1. To measure p-S6, two antibodies are available: p-S6 (Ser240/244) and p-S6 (Ser235/236). Prasse et al. used Ser235/236 for their study. Previous studies have shown that, unlike Ser240/244, Ser235/236 can be phosphorylated independently of the mTORC1 signaling pathway [22,23]. This may be a possible explanation for the difference observed in our data. Additionally, we looked at mRNA expression and not directly at the protein concentration, which may also give different results. Further studies are clearly needed, but this could open new clinical possibilities for the mTORC1 signaling pathway and its downstream proteins to be used to monitor disease activity or response to treatment.

In future studies, in our opinion, it should be assessed whether inflammatory pathways in tissue can be correlated with the same inflammatory pathways in peripheral blood to increase clinical applicability. In other diseases, for example, STAT activation already has been assessed in peripheral blood using fluorescence-activated cell sorting (FACS) [24].

There are certain limitations in our study. Firstly, the nonhomogeneous staining in tissue may lead to misinterpretation. To overcome this problem, we focused on the cells which were part of the granuloma when scoring the samples. Secondly, the study has a retrospective design. The majority of the tissue sections were originally from other hospitals in the Netherlands; therefore, not all patient characteristic information was available. Thirdly, biopsy staining only gives information regarding one time point of the disease; therefore, the following disease course cannot be visualized by this approach. Gathering information about pathway activity at different time points during disease course would yield more relevant information on the role of the signaling pathways in the pathogenesis of sarcoidosis. Fourthly, the majority of our cohort had a more severe form of sarcoidosis, resulting in an over-representation of the high COS score group. This makes it difficult to conclude whether disease status alone, irrespective of the use of medication, was associated with the activation of NLRP3. Furthermore, by dividing the COS into two groups, “resolved” (COS 1–6) and persistent (COS 7–9), the clinical assessment was limited. The fifth point to address is that we were not able to detect caspase-1 expression in sarcoid granuloma using immunohistochemistry due to the lack of a specific antibody that would give reliable staining results in formalin-fixed tissues. Finally, looking at the overall RNA expression of the signaling pathways in BALF, one can question whether this was the best method to measure the activity of the signaling pathways in BALF.

## 4. Materials and Methods

### 4.1. Study Cohort

Unstained tissue blocks of sarcoidosis patient cohorts previously studied at the St. Antonius Hospital (Nieuwegein, The Netherlands) were requested [25]. For the analysis of the signaling pathways in BALF, a new and different cohort of 25 Dutch sarcoidosis patients was included. For this cohort, it was important that leftover BALF was still present in our biobank and that the biopsy and BAL were performed on the same date. The diagnosis of sarcoidosis had been established according to the criteria of the American Thoracic Society/European Respiratory Society [1]. Patients were included in the study when residual tissue was available and when granulomas were present in the hematoxylin and eosin (HE)-stained tissue sections. The biopsies contained lung (46 samples), lymph nodes (30 samples), skin (20 samples), bone marrow (2 samples), salivary gland (1 sample), and nasal concha (1 sample) tissue. The study was approved by the Medical Research Ethics Committees United (MEC-U) of the St. Antonius Hospital (R05-08A), and written consent was obtained from all patients.

### 4.2. Immunohistochemistry

Immunohistochemical staining procedure, as well as the used antibody for mTORC1 staining, was previously described in [7]. In order to study the role of the JAK/STAT signaling pathway in granuloma formation and pathogenesis of sarcoidosis, we performed immunohistochemical staining of the activated form of STAT1 and STAT3, phosphorylated STAT1 (p-STAT1) and p-STAT3 (Cell Signaling Technology, Inc., Danvers, MA, USA). The p-STAT1 antibody was used in a concentration of 1:800 and p-STAT-3 in a concentration of 1:200 (diluted with Ventana antibody diluent, REF 251-018, Ventana Medical Systems, Inc., Tucson, AZ, USA). For the inflammasome, anti-NLRP3 was used (Thermo Scientific, Waltham, MA, USA). The anti-NLRP3 antibody was used in a concentration of 1:600 (diluted with Ventana antibody diluent, REF 251-018, Ventana Medical Systems, Inc., Tucson, AZ, USA). To identify how the inflammatory signaling pathways play a role in granuloma formation, a double immunohistochemical (IHC) staining was performed, combining the antibodies of the inflammatory signaling pathways with a marker for macrophages (CD68 (KP-1) REF 790-2931, Ventana Medical Systems, Inc., Tucson, AZ, USA), T-cells (CD3 (2GV6) REF 790-4341, Ventana Medical Systems, Inc., Tucson, AZ, USA), and B-cells (CD20 (L26) REF 760-2531, Ventana Medical Systems, Inc., Tucson, AZ, USA).

### 4.3. Evaluation of Immunohistochemistry

Supplementary Figure S1 shows the different intensities of the immunohistochemical (IHC) staining for p-S6, p-STAT1, p-STAT3, and NLRP3 in sarcoidosis patients. A pathologist with experience in ILD (CA) analyzed the results of the IHC staining. The IHC score, a grading system which is based on the percentage of antibody-positive cells within granulomas and their intensity [26], was calculated. Staining intensity was scored as 0 (negative), 1 (weak), 2 (moderate), or 3 (strong). The percentage of cells positive for the antibody was scored as 0 (<10%), 1 (10–25%), 2 (25–50%), 3 (50–75%), or 4 (>75%). The IHC score was generated as the sum of intensity and percentage of positive cells, ranging from 0 to 7.

### 4.4. Clinical Characteristics

To determine possible related characteristics to active signaling pathways, basic characteristics (age at diagnosis, gender, comorbidities as reported in the medical records), Scadding stage at diagnosis and follow-up, presence of Löfgren syndrome, and therapy, as well as organ manifestation, were collected from medical records of patients. The disease status of patients was retrospectively examined and classified according to the Clinical Outcome Status (COS) [27] 2 and 5 years after diagnosis. COS categorizes patients into 9 scores: (1) resolved disease, never treated, (2) resolved disease, no therapy > one year, (3) minimal disease, never treated, (4) minimal disease, no therapy > one year, (5) persistent disease, never treated, (6) persistent disease, no therapy > one year, (7) persistent disease, current therapy but no worsening in prior year and asymptomatic, (8) persistent disease, current therapy but no worsening in prior year and symptomatic, and (9) persistent disease, current therapy which worsened in the prior year. Sarcoidosis patients were classified into two phenotype groups: resolved, minimal, or persistent disease without treatment (COS 1–6) and persistent disease with need for treatment (COS 7–9) [25].

### 4.5. RNA Isolation and Quantitative Real-Time PCR (qPCR)

RNA expression of the signaling pathways was measured using qPCR in cells derived from bronchoalveolar lavage fluid (BALF) taken on the same date as the biopsy material. Forty Dutch sarcoidosis patients with biopsy and BALF were selected. Total RNA was isolated using TRIzol-LS according to manufacturer’s protocol. RNA concentration and purity were determined using NanoDrop. cDNA synthesis using an I-script cDNA synthesis kit (bio-RAD, Veenendaal, The Netherlands) was performed as per manufacturers instruction. qPCR was performed on Quantstudio 5. Briefly, the reaction mixture consisted of 10 ng cDNA, 2x POWER SYBR Green master mix (Applied Biosystem, Foster City, CA, USA) and 5 μM forward and reverse primers. Thermal cycling conditions were 2 min at 95 °C, followed by 45 cycles of 10 sec at 95 °C, 20 sec at 61 °C, and 25 sec at 72 °C. Melt curve analysis was employed at the end of each PCR to confirm the specificity of the PCR product. Each measurement was performed in duplicate. Relative RNA expression was calculated using the delta Ct method [28], using the mean of three housekeeping genes, Eukaryotic translation elongation factor 1 alpha 1 (EEF1A1), beta-2-microglobulin (B2M), and β-actin and multiplied by 100. All PCR primers were synthesized by Sigma-Aldrich (St. Louis, MI, USA) and crosschecked using BLAST sequence analysis (NCBI, Bethesda, MD, USA). The sequences of all primers used in this study are listed in Table 2. RNA expression was investigated for genes involved in the mTOR (mTORC1 and S6K1), JAK/STAT (JAK1, JAK2, STAT1, and STAT3), and NLRP3 (NLRP3 and caspase-1) signaling pathways.

### 4.6. Statistical Analysis

Data were analyzed using IBM SPSS statistics version 24.0 (IMB Corp, Armonk, NY, USA). An unpaired T-test was used to compare numerical data. Nonparametric tests were used for non-normally distributed data (Mann–Whitney U test). Spearman’s rank correlation coefficients (expressed as r) were used to assess associations between two variables. Multiple linear regression models were used to assess associations between continuous variables. Log transformation was used for non-normally distributed variables. All tests were two-sided, and a *p*-value of 0.05 indicated significance.

## 5. Conclusions

The inflammatory pathways NLRP3, JAK-STAT, and mTORC1 are not simultaneously active in all patients’ sarcoidosis at diagnosis. The activation of the NLRP3 inflammasome pathway was present in 85% of patients with sarcoidosis at diagnosis, and its presence is associated with a worse clinical outcome of the disease. The activation of mTORC1 and JAK/STAT was seen in 50% of patients at diagnosis and was not associated with disease course. Our finding of different new conceptual inflammatory tissue phenotypes in sarcoidosis could possibly guide future treatment studies using the available inhibitors of either NLRP3, JAK-STAT, and mTORC1 in a more personalized medicine approach.

## Figures and Tables

**Table 1:** Immunohistochemical (IHC) scoring for p-S6, p-STAT1, p-STAT3, and NLRP3. Addition of the percentage of positive cells within the granuloma (0–4) and staining intensity (0–3) results in the ranking from 0 to 7. Data are shown as absolute numbers, with percentages between brackets.

IHC Score	p-S6 *n* (%)	p-STAT1	p-STAT3	NLRP3
0	29 (49.2)	30 (50.0)	44 (73.3)	9 (15.0)
1	13 (22.0)	13 (21.7)	9 (15.0)	4 (6.7)
2	7 (11.9)	5 (8.3)	5 (8.3)	4 (6.7)
3	6 (10.2)	2 (3.3)	0 (0.0)	6 (10.0)
4	4 (6.8)	3 (5.0)	2 (3.3)	5 (8.3)
5	0 (0)	3 (5.0)	0 (0.0)	2 (3.3)
6	0 (0)	1 (1.7)	0 (0.0)	14 (23.3)
7	0 (0)	3 (5.0)	0 (0.0)	16 (26.7)

**Table 2:** Specific forward and reverse primers for analysis of mRNA levels using real-time PCR.

Gene	Forward	Reverse
mTORC1 [29]	*5′* *GGAGGCTGATGGACACAAAT 3′*	*5′* *CTGTGGTCCCCGTTTTCTTA 3′*
S6K1 [30]	*5′* *CTCTGAGGATGAGCTGGAGG* *3′*	*5′* *TTCTCACAATGTTCCATGCC* *3′*
JAK1 *	*5′* *CACAGGCATGCCGTATCTCT 3′*	*5′* *CCAGAGCTTGGTGTTCTCGT 3′*
JAK2 [31]	*5′* *GATGGATGCCCAGATGAGAT 3′*	*5′* *TTGATCCACTCGAAGAGCTAGA 3′*
STAT1 [32]	*5′* *ACCTAACGTGCTGTGCGTAG* *3′*	*5′* *GAGACATCCTGCCACCTTGT* *3′*
STAT3 [33]	*5′* *GCTTCCTGCAAGAGTCGAAT* *3′*	*5′* *ATTGGCTTCTCAA GATACCTG* *3′*
NLRP3 [34]	*5′ AAGCCAAGAATCCACAGTGTAAC 3′*	*5′* *TTGCCTCGCAGGTAAAGGT 3′*
Caspase-1 [35]	*5′* *CCTTAATATGCAAGACTCTCAAGGA* *3′*	*5′* *TAAGCTGGGTTGTCCTGCACT* *3′*

mTORC1, mechanistic target of rapamycin complex 1; S6K1, target protein S6 kinase-1; JAK1, Janus kinase-1; JAK2, Janus kinase-2; STAT1, signal transducers and activators of transcription-1; STAT3, signal transducers and activators of transcription; NLRP3, NLR family pyrin domain-containing-3. Superscript numerals denote reference numbers; * designed by authors.

## Data Availability

The data presented in this study are available on request from the corresponding author.

## Supplementary Materials

The supporting information can be downloaded at: https://www.mdpi.com/article/10.3390/ijms241612792/s1.

## References

[1] Costabel U., Hunninghake G.W. (1999). ATS/ERS/WASOG statement on sarcoidosis. Sarcoidosis Statement Committee. American Thoracic Society. European Respiratory Society. World Association for Sarcoidosis and Other Granulomatous Disorders. Eur. Respir. J..

[2] Broos C.E., Koth L.L., Van Nimwegen M., In’T Veen J.C.C.M., Paulissen S.M.J., Van Hamburg J.P., Annema J.T., Heller-Baan R., Kleinjan A., Hoogsteden H.C. (2018). Increased T-helper 17.1 cells in sarcoidosis mediastinal lymph nodes. Eur. Respir. J..

[3] Zhou T., Casanova N., Pouladi N., Wang T., Lussier Y., Knox K.S., Garcia J.G.N.N. (2017). Identification of Jak-STAT signaling involvement in sarcoidosis severity via a novel microRNA-regulated peripheral blood mononuclear cell gene signature. Sci. Rep..

[4] Gerke A.K. (2020). Treatment of Sarcoidosis: A Multidisciplinary Approach. Front. Immunol..

[5] Riteau N., Bernaudin J.F. (2020). In addition to mTOR and JAK/STAT, NLRP3 inflammasome is another key pathway activated in sarcoidosis. Eur. Respir. J..

[6] Linke M., Pham H.T.T., Katholnig K., Schnöller T., Miller A., Demel F., Schütz B., Rosner M., Kovacic B., Sukhbaatar N. (2017). Chronic signaling via the metabolic checkpoint kinase mTORC1 induces macrophage granuloma formation and marks sarcoidosis progression. Nat. Immunol..

[7] Kraaijvanger R., Seldenrijk K., Beijer E., Damen J., Wilson J.L., Weichhart T., Grutters J.C., Veltkamp M. (2021). Activation of downstream mTORC1 target ribosomal protein S6 kinase (S6K) can be found in a subgroup of Dutch patients with granulomatous pulmonary disease. Cells.

[8] Gupta N., Bleesing J.H., McCormack F.X. (2020). Successful response to treatment with sirolimus in pulmonary sarcoidosis. Am. J. Respir. Crit. Care Med..

[9] Damsky W., Thakral D., Emeagwali N., Galan A., King B. (2018). Tofacitinib treatment and molecular analysis of cutaneous sarcoidosis. N. Engl. J. Med..

[10] Zhou E.R., Arce S. (2020). Key players and biomarkers of the adaptive immune system in the pathogenesis of sarcoidosis. Int. J. Mol. Sci..

[11] Damsky W., Wang A., Kim D.J., Young B.D., Singh K., Murphy M.J., Daccache J., Clark A., Ayasun R., Ryu C. (2022). Inhibition of type 1 immunity with tofacitinib is associated with marked improvement in longstanding sarcoidosis. Nat. Commun..

[12] Kerkemeyer K.L., Meah N., Sinclair R.D. (2021). Tofacitinib for cutaneous and pulmonary sarcoidosis: A case series. J. Am. Acad. Dermatol..

[13] Friedman M.A., Le B., Stevens J., Desmarais J., Seifer D., Ogle K., Choi D., Harrington C.A., Jackson P., Rosenbaum J.T. (2021). Tofacitinib as a Steroid-Sparing Therapy in Pulmonary Sarcoidosis, an Open-Label Prospective Proof-of-Concept Study. Lung.

[14] Huppertz C., Jäger B., Wieczorek G., Engelhard P., Oliver S.J., Bauernfeind F.G., Littlewood-Evans A., Welte T., Hornung V., Prasse A. (2020). The NLRP3 inflammasome pathway is activated in sarcoidosis and involved in granuloma formation. Eur. Respir. J..

[15] Zhang H., Costabel U., Dai H. (2021). The Role of Diverse Immune Cells in Sarcoidosis. Front. Immunol..

[16] Wu B., Sodji Q.H., Oyelere A.K. (2022). Inflammation, Fibrosis and Cancer: Mechanisms, Therapeutic Options and Challenges. Cancers.

[17] Li X., Jiang M., Chen X., Sun W. (2022). Etanercept alleviates psoriasis by reducing the Th17/Treg ratio and promoting M2 polarization of macrophages. Immun. Inflamm. Dis..

[18] Xia T., Zhang M., Lei W., Yang R., Fu S., Fan Z., Yang Y., Zhang T. (2023). Advances in the role of STAT3 in macrophage polarization. Front. Immunol..

[19] Artlett C.M., Sassi-Gaha S., Rieger J.L., Boesteanu A.C., Feghali-Bostwick C.A., Katsikis P.D. (2011). The Inflammasome Activating Caspase 1 Mediates Fibrosis and Myofibroblast Differentiation in Systemic Sclerosis. ARTHRITIS Rheum..

[20] Lasithiotaki I., Giannarakis I., Tsitoura E., Samara K.D., Margaritopoulos G.A., Choulaki C., Vasarmidi E., Tzanakis N., Voloudaki A., Sidiropoulos P. (2016). NLRP3 inflammasome expression in idiopathic pulmonary fibrosis and rheumatoid lung. Eur. Respir. J..

[21] Prasse A., Artysh N., Culver D.A., Young P.R., Jäger B. (2023). High MTOR Activity in Bronchoalveolar Lavage Cells From Patients with Sarcoidosis. Am. J. Respir. Crit. Care Med..

[22] Roux P.P., Shahbazian D., Vu H., Holz M.K., Cohen M.S., Taunton J., Sonenberg N., Blenis J. (2011). RAS/ERK Signaling Promotes Site-specific Ribosomal Protein S6 Phosphorylation via RSK and Stimulates Cap-dependent Translation. J. Biol. Chem..

[23] Pende M., Um S.H., Mieulet V., Sticker M., Goss V.L., Mestan J., Mueller M., Fumagalli S., Kozma S.C., Thomas G. (2004). S6K1 −/−/S6K2 −/− Mice Exhibit Perinatal Lethality and Rapamycin-Sensitive 5′-Terminal Oligopyrimidine mRNA Translation and Reveal a Mitogen-Activated Protein Kinase-Dependent S6 Kinase Pathway. Mol. Cell Biol..

[24] Canto E., Isobe N., Didonna A., Hauser S.L., Oksenberg J.R., Baranzini S., Bevan C., Bove R., Crabtree-Hartman E., Gelfand J.M. (2018). Aberrant STAT phosphorylation signaling in peripheral blood mononuclear cells from multiple sclerosis patients. J. Neuroinflammation.

[25] Beijer E., Seldenrijk K., Meek B., Damen J., Quanjel M.J.R., Grutters J.C., Veltkamp M. (2021). Detection of C. acnes in granulomas of patients with either hypersensitivity pneumonitis or vasculitis reveals that its presence is not unique for sarcoidosis. ERJ Open Res..

[26] Gulhati P., Cai Q., Li J., Liu J., Rychahou P.G., Qiu S., Lee E.Y., Silva S.R., Bowen K.A., Gao T. (2009). Targeted inhibition of mammalian target of rapamycin signaling inhibits tumorigenesis of colorectal cancer. Clin. Cancer Res..

[27] Baughman R.P., Nagai S., Balter M., Costabel U., Drent M., Du Bois R., Grutters J.C., Judson M.A., Lambin I., Lower E.E. (2011). Defining the clinical outcome status (COS) in sarcoidosis: Results of WASOG Task Force. Sarcoidosis Vasc. Diffus. Lung Dis..

[28] Livak K.J., Schmittgen T.D. (2001). Analysis of relative gene expression data using real-time quantitative PCR and the 2-ΔΔCT method. Methods.

[29] Treeck O., Wackwitz B., Haus U., Ortmann O. (2006). Effects of a combined treatment with mTOR inhibitor RAD001 and tamoxifen in vitro on growth and apoptosis of human cancer cells. Gynecol. Oncol..

[30] Berman A.Y., Manna S., Schwartz N.S., Katz Y.E., Sun Y., Behrmann C.A., Yu J.J., Plas D.R., Alayev A., Holz M.K. (2017). ERRα regulates the growth of triple-negative breast cancer cells via S6K1-dependent mechanism. Signal Transduct. Target. Ther..

[31] Lee K.-Y., Lee Y.-L., Chiang M.-H., Wang H.-Y., Chen C.-Y., Lin C.-H., Chen Y.-C., Fan C.-K., Cheng P.-C. (2021). Schistosoma egg antigens suppress LPS-induced inflammation in human IMR-90 cells by modulation of JAK/STAT1 signaling. Immunol. Infect..

[32] Najafi S., Saadat P., Beladi Moghadam N., Manoucherinia A., Aghazadeh Z., Vali Mohammadi A., Noorbakhsh S.M., Movahedi M., Nikouei Moghaddam M.R., Pashaiefar H. (2022). The Effects of Mannuronic Acid on IL-1β, IL-17A, STAT1, and STAT3 Gene Expressions and TLR2 and TLR4 Molecules in Multiple Sclerosis. J. Clin. Pharmacol..

[33] Ganji P.N., Park W., Wen J., Mahaseth H., Landry J., Farris A.B., Willingham F., Sullivan P.S., Proia D.A., El-Hariry I. (2013). Antiangiogenic effects of ganetespib in colorectal cancer mediated through inhibition of HIF-1α and STAT-3. Angiogenesis.

[34] Shi X., Xie W.L., Kong W.W., Chen D., Qu P. (2015). Expression of the NLRP3 Inflammasome in Carotid Atherosclerosis. J. Stroke Cerebrovasc. Dis..

[35] Ran S., Liu B., Gu S., Sun Z., Liang J. (2017). Analysis of the expression of NLRP3 and AIM2 in periapical lesions with apical periodontitis and microbial analysis outside the apical segment of teeth. Arch. Oral. Biol..

