# Supplementary material for: Simultaneous Assessment of mTORC1, JAK/STAT, and NLRP3 Inflammasome Activation Pathways in Patients with Sarcoidosis
Source: Int J Mol Sci. 2023 Aug 14;24(16):12792. doi: 10.3390/ijms241612792 (PMC10454122; doi:10.3390/ijms241612792)
Supplement: Supplementary file 1 [file ijms-24-12792-s001.zip › Supplementary Figure S1.pdf]

International Journal of *Molecular Sciences*

MDPI

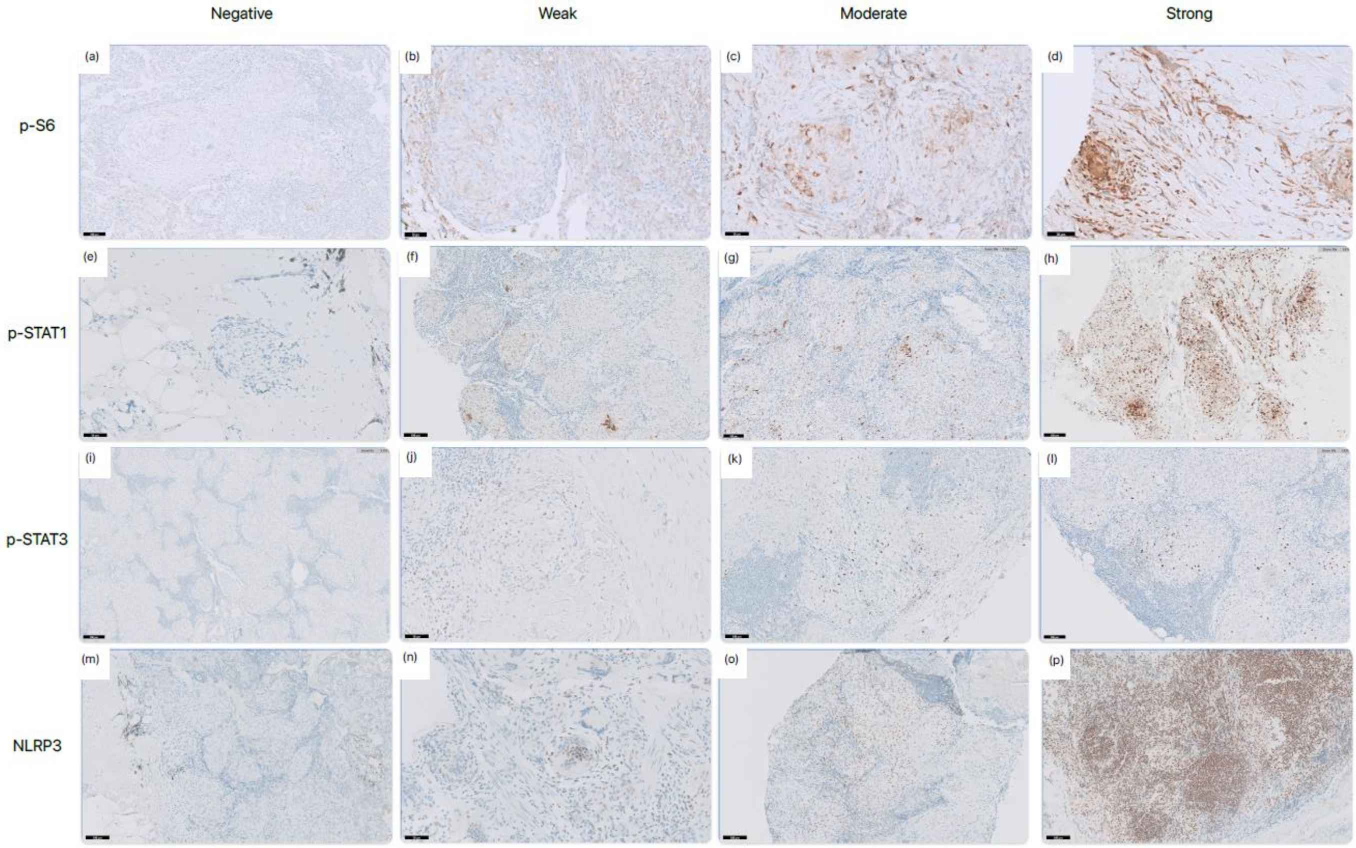

Figure S1. immunohistochemistry (IHC) staining of the three key inflammatory signaling pathways in sarcoid tissue. a) – d) sarcoid tissue was immunohistochemically stained using p-S6 antibodies. e) – h) activated STAT-1, i) – l) activated STAT3 and m) – p) NLRP3. a), e), i), m) represent negative staining, b), f), j), n) represent weak staining, c), g), k), o) represent moderate staining, and d), h), l), p) represent strong staining. Scale bars = 200 µm (i) 100 µm (a, f, g, h, k, l, m, o, p), and 50 µm (b, c, d, e, j, n).
