# Supplementary material for: Simultaneous Assessment of mTORC1, JAK/STAT, and NLRP3 Inflammasome Activation Pathways in Patients with Sarcoidosis
Source: Int J Mol Sci. 2023 Aug 14;24(16):12792. doi: 10.3390/ijms241612792 (PMC10454122; doi:10.3390/ijms241612792)
Supplement: Supplementary file 1 [file ijms-24-12792-s001.zip › Supplementary table S1.pdf]

International Journal of
Molecular Sciences

MDPI

Table S1. Characteristics of sarcoidosis patients. Data is shown as absolute numbers and percentage.

**Parameter**		***n***	
**Age[a] (years)**		60	43.5 ± 12.1
**Male sex**		32/60	46.7%
**Ever smoker**		33/60	55.9%
**Caucasian**		49/60	81.7%
**Löfgren syndrome**		2/60	3.4%
**Medication at time of biopsy**		7/60	11.7%
**Third-line therapy[b]**		24/60	40.0%
**COS group 2 years follow up**		55/60	
Group 1-6		17	30.9%
Group 7-9		38	69.1%
**COS group 5 years follow up**		47/60	
Group 1-6		18	38.3%
Group 7-9		29	61.7%
**Scadding stage at time biopsy**		42/60	
Stage 0		1	2.4%
Stage I		13	31%
Stage II		16	38.1%
Stage III		7	16.7%
Stage IV		5	11.9%
**Inflammatory biomarkers[c]**	*Ref.range*		
ACE (U/L)	*12-70*	19/60	46.6 ± 34.9
sIL-2R (pg/mL)	*<3000*	14/60	5133.2 ± 3028.6
**Extra pulmonary involvement**		41/60	68.3%
Skin		21/60	35.0%
Eyes		4/60	6.7%
Liver		7/60	11.7%
Heart		9/60	15%
Spleen		2/60	3.3%
Bones		4/60	6.7%
Nerve system		11/60	18.3%
SFN		7/60	11.7%

[a]Age at time of biopsy and is shown as mean ± SD. [b]Third-line therapy consisted of infliximab use at follow up. [C]biomarkers at time of diagnosis and is shown as mean ± SD. COS: Clinical outcome status, SFN: Small fiber neuropathy, Scadding stages: 0 = Normal chest radiograph; I = Bilateral hilar lymphadenopathy (BHL); II = BHL with pulmonary infiltrates; III = pulmonary infiltrates without BHL; IV = fibrosis.
